# Socio-economic and lifestyle factors associated with the risk of prostate cancer

**DOI:** 10.1054/bjoc.1999.1105

**Published:** 2000-03-06

**Authors:** T I Lund Nilsen, R Johnsen, L J Vatten

**Affiliations:** Department of Community Medicine and General Practice, Norwegian University of Science and Technology, University Medical Centre, Trondheim, N-7489, Norway; Norwegian Cancer Society, PO Box 5327 Majorstua, Oslo, N-0304, Norway

**Keywords:** prostate cancer, risk, socio-economic factors, lifestyle

## Abstract

International and interethnic differences in prostate cancer incidence suggest an environmental aetiology, and lifestyle and socio-economic factors have been studied, but with divergent results. Information on a cohort of 22 895 Norwegian men aged 40 years and more was obtained from a health examination and two self-administered questionnaires. Information on incident cases of prostate cancer was made available from the Cancer Registry. We used the Cox proportional hazards model to calculate incidence rate ratios as estimates of the relative risk (RR) with 95% confidence interval (CI). Reported *P* -values are two-sided. During a mean follow-up of 9.3 years, 644 cases were diagnosed. Risk was elevated among men in occupations of high compared to low socio-economic status (RR = 1.30; 95% CI 1.05–1.61), and among men with high education compared to the least educated (RR = 1.56; 95% CI 1.11–2.19). A RR of 1.56 (95% CI 0.97–2.44) suggests a higher risk among divorced or separated men, compared with married men. We also found indications of a weak negative association with leisure-time physical activity (RR = 0.80; 95% CI 0.62–1.03 for high vs low activity), a weak positive association with increasing number of cigarettes (*P* = 0.046), while alcohol consumption was not related to the risk of prostate cancer. These results show that high socio-economic status is associated with increased risk of prostate cancer, and that divorced or separated men might be at higher risk than married men. Data from this study also indicate that high levels of physical activity may reduce prostate cancer risk. © 2000 Cancer Research Campaign


*British Journal of Cancer* (2000) **82**(7), 1358–1363
© 2000 Cancer Research Campaign
DOI: 10.1054/ bjoc.1999.1105, available online at http://www.idealibrary.com on IDEAL®

### Socio-economic and lifestyle factors associated with the risk of prostate cancer

**TI Lund Nilsen[1,2], R Johnsen[1] and LJ Vatten[1]**

[1]Department of Community Medicine and General Practice, Norwegian University of Science and Technology, University Medical Centre, N-7489 Trondheim, Norway; [2]Norwegian Cancer Society, PO Box 5327 Majorstua, N-0304 Oslo, Norway.

**Summary** International and interethnic differences in prostate cancer incidence suggest an environmental aetiology, and lifestyle and socio-economic factors have been studied, but with divergent results. Information on a cohort of 22 895 Norwegian men aged 40 years and more was obtained from a health examination and two self-administered questionnaires. Information on incident cases of prostate cancer was made available from the Cancer Registry. We used the Cox proportional hazards model to calculate incidence rate ratios as estimates of the relative risk (RR) with 95% confidence interval (CI). Reported *P*-values are two-sided. During a mean follow-up of 9.3 years, 644 cases were diagnosed. Risk was elevated among men in occupations of high compared to low socio-economic status (RR = 1.30; 95% CI 1.05–1.61), and among men with high education compared to the least educated (RR = 1.56; 95% CI 1.11–2.19). A RR of 1.56 (95% CI 0.97–2.44) suggests a higher risk among divorced or separated men, compared with married men. We also found indications of a weak negative association with leisure-time physical activity (RR = 0.80; 95% CI 0.62–1.03 for high vs low activity), a weak positive association with increasing number of cigarettes ($P = 0.046$), while alcohol consumption was not related to the risk of prostate cancer. These results show that high socio-economic status is associated with increased risk of prostate cancer, and that divorced or separated men might be at higher risk than married men. Data from this study also indicate that high levels of physical activity may reduce prostate cancer risk. © 2000 Cancer Research Campaign

**Keywords:** prostate cancer; risk; socio-economic factors; lifestyle

Prostate cancer is a common malignancy in Western societies, and a leading cause of male cancer deaths (Cancer Registry of Norway, 1998; Landis et al, 1998), but the current knowledge of its aetiology is meagre. Nonetheless, the international variation (Muir et al, 1991; Parker et al, 1998), and especially that risk is modified by migration (Haenszel and Kurihara, 1968), indicates environmental mechanisms of causation.

Cigarette smoking is generally not considered a risk factor for prostate cancer (Colditz, 1996), although a few studies have reported a positive association (Hsing et al, 1991; Cerhan et al, 1997). Similarly, there are conflicting findings on the association between alcohol consumption and the risk of prostate cancer (Hiatt et al, 1994; Andersson et al, 1996; Hayes et al, 1996; Lumey et al, 1998). Available results for physical activity and fitness are inconsistent (Le Marchand et al, 1991; Lee et al, 1992; Thune and Lund, 1994; Hartman et al, 1998), as are those considering marital status and socio-economic factors (Talamini et al, 1986; Severson et al, 1989; Hayes et al, 1992; Andersson et al, 1996; Harvei and Kravdal, 1997). A possible relation between diabetes and prostate cancer has been investigated under the hypothesis that an altered endocrine milieu in men with this disease protects against prostate cancer, but findings are inconclusive (Steenland et al, 1995; Giovannucci et al, 1998).

*Received 16 August 1999*
*Revised 27 September 1999*
*Accepted 27 September 1999*

*Correspondence to*: TI Lund Nilsen, Department of Community Medicine and General Practice, University Medical Centre, N-7489 Trondheim, Norway

Using prospective data from a large health screening survey, we examined the association between several lifestyle and socio-economic factors and the development of prostate cancer in a cohort of Norwegian men.

### MATERIALS AND METHODS

### The cohort

From 1984 to 1986, the National Health Screening Service in Norway conducted a large health survey in the county of Nord-Trøndelag, where all residents aged 20 years or more by 31 December 1983 were invited to participate. Among 85 100 eligible persons, 77 310 (90.8%) filled in the questionnaire that was mailed with the invitation. The health examination included measurements of height and weight, capillary blood glucose level, blood pressure and pulse rate. A second questionnaire was handed out together with a pre-stamped envelope that the participants were asked to fill in and return from home. Among other items, this second questionnaire included detailed questions on smoking history, alcohol consumption, physical activity, educational attainment and occupation. A more comprehensive description of the participants, questionnaires and screening procedures is previously given by Holmen and Midthjell (1990).

We restricted the study to men who were at risk of developing the disease, and included all 22 895 men aged 40 years or more at baseline who had no history of any cancer at study entry. Each participant contributed person-years from the date of study entry until the date of a cancer diagnosis (of all sites), death, emigration, or the cut-off date of 1 January 1996, whichever occurred first.

### Follow-up

Every citizen in Norway is given a unique 11-digit identity number, and Norwegian law strictly regulates the use of this number. The Norwegian Data Inspectorate, the Norwegian Board of Health, and the Regional Committee for Ethics in Medical Research approved our study protocol. Being attached to each participant's record, the identity number enabled linkage to the Norwegian Cancer Registry in order to identify incident cases of prostate cancer that occurred in the cohort during follow-up.

Mandatory reporting of cancer is regulated by Norwegian law, and the Cancer Registry has since 1953 registered all incident cases of cancer in Norway. To achieve a high degree of completeness and high data quality, the material of the Cancer Registry is matched against the Register of Deaths at Statistics Norway. For all cases registered since 1953, 84.7% are histologically verified, and only 1.7% of the diagnoses are based on death certificate alone (Cancer Registry of Norway, 1998). Evaluation of the quality of prostate cancer data in the Cancer Registry has shown a completeness of nearly 100% (Harvei et al, 1996).

### Study factors

Exposure data was collected from three sources. Marital status was added to the health survey database from Statistics Norway, clinical data were obtained at the physical examination included in the screening programme, and the questionnaires provided information on lifestyle and demographic characteristics. We classified smoking status in three categories, where individuals who had never smoked cigarettes daily were considered non-smokers, and men who reported previous or present daily smoking were classified as former and current smokers respectively. We also analysed the association with number of cigarettes smoked per day (in quartiles) and pack-years of smoking (in quartiles), using non-smokers as the reference. For alcohol consumption, the reference category included men not drinking alcohol the past 2 weeks, whereas men who reported drinking 1–4 times and more than 4 times the past 2 weeks comprised category two and three. Moreover, men who reported to be teetotallers were compared to drinking men, and men who reported periods of excessive drinking were compared to men without such periods.

In the health survey questionnaire on leisure-time physical activity, the participants where asked, 'how often do you exercise?', 'how hard do you exercise?', and "for how long do you carry on?', with five, three and four response options respectively. Regarding frequency of leisure-time physical activity, we considered men who exercised less than once a week as inactive, whereas individuals exercising 1–3 times per week and more than 3 times per week were classified as moderately active and highly active. In addition, we utilized the information on frequency, intensity and duration to calculate a summary measure (index) of physical activity, categorized into three equal groups labelled low, medium and high. Occupational physical activity was classified according to how often they reported being physically worn out after a day's work (low activity when reporting almost never or infrequently worn out and high activity when reporting often or nearly always worn out). For the association with cardiovascular fitness, we analysed pulse rate and systolic blood pressure in quartiles, however, the results are presented as dichotomized variables. History of diabetes mellitus (both insulin-dependent and non-insulin-dependent) was assessed from the baseline questionnaire, whereas levels of non-fasting blood glucose among non-diabetics was dichotomized according to the cut point for a 'positive screening' used in the health survey (≥ 8.0 mmol/l) (Holmen and Midthjell, 1990), which is in conformity with the WHO criteria of 1980 (WHO, 1980).

Marital status was classified as married, unmarried, widower and divorced/separated. Educational attainment was classified as primary and lower secondary school (0–9 years), upper secondary school (10–12 years) and college or university (>12 years). Finally, different occupational categories with respect to socio-economic status were classified as follows: unemployed men, unskilled manual workers, fishermen and subordinate staff comprised the reference category, while category two included men occupied in farming, agriculture, or forestry. The third category (i.e. occupations of highest socio-economic status) included skilled manual workers, men in professional or management positions and self-employed individuals.

### Statistical analysis

The Cox proportional hazards model (Kleinbaum, 1995) was used to calculate relative risk estimates for each of the variables under study. Because prostate cancer is highly age-dependent, we included age (across 2.5-year categories) and the relevant exposure variable as independent variables, and individual number of person-years as the dependent variable. This produced age-adjusted incidence rate ratios as estimates of the relative risk (RR) with 95% confidence interval (CI). When appropriate, a two-sided test for trend across exposure categories was calculated by treating the categories as ordinal variables in the proportional hazards model.

On the basis of the results thus obtained, we evaluated potential confounding between each exposure variable and other available factors. Thus, the multivariate analysis included cigarettes per day, alcohol consumption, leisure-time physical activity index, marital status, educational attainment and occupation. Due to inter-correlation between educational attainment and occupation, these variables were not treated simultaneously in the multivariate analysis.

In a separate analysis, we used 1 January 1993 as cut-off date for follow-up, in order to avoid potential bias due to differential testing with prostate-specific antigen (PSA) between categories of marital status, education and occupation. We also conducted a separate analysis for metastatic prostate cancer, since the risk of localized and invasive prostate cancer may vary with respect to the variables under study. Due to small numbers with metastatic prostate cancer we analysed some of the variables using fewer categories than in the previous analysis, and adjustment for age was made using 5-year categories.

All statistical analyses were performed using the statistical software SPSS for Windows (Release 8.0.0, Copyright © SPSS Inc. 1989–1997).

### RESULTS

A total of 644 men were diagnosed with prostate cancer (mean follow-up = 9.3 years), and 212 720 person-years were observed altogether. Mean age at diagnosis was 75.5 years (range 49.2–96.2

© 2000 Cancer Research Campaign

**Table 1** Follow-up study of 22 895 Norwegian men participating in a large health survey. Number of individuals included between 1984 and 1986 and accumulated person years during 12 years of follow-up,[a] incident cases and incidence rate of prostate cancer by age at entry

| Age at entry (years) | No. of men (% of total) | No. of person years (% of total) | No. of cases (% within age-group) | Incidence rate (cases/10 000 py) |
|---|---|---|---|---|
| 40–49 | 6133 (26.8) | 65 432 (30.8) | 7 (0.1) | 1.1 |
| 50–59 | 5540 (24.2) | 56 882 (26.7) | 78 (1.4) | 13.7 |
| 60–69 | 6034 (26.3) | 55 086 (25.9) | 229 (3.8) | 41.6 |
| 70–79 | 3818 (16.7) | 28 328 (13.3) | 257 (6.7) | 90.7 |
| ≥80 | 1370 (6.0) | 6992 (3.3) | 73 (5.3) | 104.4 |

[a]Censored by death, cancer diagnosis (all sites) and migration.

**Table 2** Age-adjusted relative risks (RRs) and 95% confidence intervals (CIs) of incident prostate cancer associated with smoking habits and alcohol consumption in a cohort of 22 895 Norwegian men

| Variable[a] | No. of cases | No. of men | No. of person-years | RR | 95% CI | *P* for trend[b] |
|---|---|---|---|---|---|---|
| Smoking status | | | | | | |
| Never | 222 | 6316 | 58 155 | 1.00 | Reference | |
| Former | 183 | 6097 | 57 748 | 0.98 | 0.80–1.19 | |
| Current | 153 | 6627 | 62 162 | 0.96 | 0.78–1.19 | 0.693 |
| Cigarettes/day | | | | | | |
| 0 | 222 | 6316 | 58 155 | 1.00 | Reference | |
| 1–8 | 73 | 2754 | 25 574 | 0.84 | 0.64–1.10 | |
| 9–10 | 67 | 2689 | 25 748 | 1.05 | 0.79–1.39 | |
| 11–15 | 51 | 2079 | 20 344 | 1.37 | 1.00–1.88 | |
| >15 | 45 | 2255 | 22 289 | 1.27 | 0.91–1.76 | 0.046 |
| Pack-years (20 cigarettes/pack) | | | | | | |
| 0 | 222 | 6316 | 58 155 | 1.00 | Reference | |
| 1–10 | 50 | 2520 | 25 064 | 0.95 | 0.70–1.30 | |
| 11–17 | 34 | 2021 | 19 850 | 0.84 | 0.58–1.21 | |
| 18–25 | 58 | 2107 | 20 334 | 1.24 | 0.92–1.67 | |
| >25 | 73 | 2241 | 20 593 | 1.22 | 0.93–1.60 | 0.102 |
| Alcohol consumption the past 2 weeks | | | | | | |
| None (but not teetotaller) | 281 | 8447 | 76 302 | 1.00 | Reference | |
| 1–4 times | 148 | 6844 | 67 939 | 1.15 | 0.94–1.41 | |
| >4 times | 40 | 1721 | 16 756 | 0.90 | 0.64–1.25 | 0.862 |
| Teetotaller | | | | | | |
| No | 469 | 17 012 | 160 998 | 1.00 | Reference | |
| Yes | 80 | 1700 | 14 445 | 1.22 | 0.96–1.55 | |
| Excessive drinking during some period(s) of life | | | | | | |
| No | 319 | 11 368 | 107 573 | 1.00 | Reference | |
| Possibly/yes | 140 | 5291 | 50 278 | 1.09 | 0.90–1.34 | |

[a]Information on each variable was not available on all participants. [b]Two-sided *P*-values for trend by proportional hazards model when variables were treated as ordinal variables.

years). The age-specific incidence rates (Table 1) correspond to those of the total Norwegian population (Cancer Registry of Norway, 1998), and the lifetime risk (up to 75 years) of prostate cancer would be 9.7%.

Table 2 shows the age-adjusted RR of prostate cancer associated with smoking habits and alcohol consumption. Smoking status and pack-years of smoking were not associated with prostate cancer risk, but there was a slightly increased risk with increasing number of cigarettes ($P = 0.046$). Frequency of alcohol consumption the past 2 weeks was not significantly associated with prostate cancer. However, comparing teetotallers to drinking men, we found a slightly increased risk among teetotallers (RR = 1.22; 95% CI 0.96–1.55). The associations with cigarette smoking and alcohol consumption were not materially changed after adjustment for potential confounding with each other or with marital status, education and occupation. Adjustment for physical activity changed the estimates only modestly (data not shown).

The results for physical activity, cardiorespiratory fitness, diabetes and blood glucose are presented in Table 3. Overall, there was no strong association, but the most physically active men had a suggestive 20% reduction in prostate cancer risk compared to the least active (RR = 0.80; 95% CI 0.62–1.03), which did not change after adjustment for potentially confounding variables (data not shown). Additionally, men with known diabetes mellitus had a relative risk of 1.31 compared to men without the disease (95% CI 0.93–1.82).

Table 4 shows that divorced or separated men might be at higher risk of prostate cancer than married men (RR = 1.56; 95% CI 0.97–2.44). Increasing years of education was positively associated with prostate cancer risk ($P = 0.007$), and men in occupations of high socio-economic status had higher risk than men in

 *© 2000 Cancer Research Campaign*

**Table 3** Age-adjusted relative risks (RRs) and 95% confidence intervals (CIs) of incident prostate cancer associated with measures of physical activity, cardiovascular fitness, diabetes, and blood glucose levels in a cohort of 22 895 Norwegian men

| Variable[a] | No. of cases | No. of men | No. of person-years | RR | 95% CI | *P* for trend[b] |
|---|---|---|---|---|---|---|
| Leisure-time physical activity | | | | | | |
| Inactive | 178 | 7385 | 68 384 | 1.00 | Reference | |
| Moderately active | 217 | 8053 | 78 205 | 1.05 | 0.86–1.28 | |
| Highly active | 136 | 3187 | 28 042 | 1.01 | 0.81–1.27 | 0.882 |
| Frequency-intensity-duration-index of leisure-time physical activity | | | | | | |
| Low | 141 | 4163 | 37 459 | 1.00 | Reference | |
| Medium | 136 | 4348 | 42 125 | 1.08 | 0.85–1.37 | |
| High | 107 | 4259 | 41 450 | 0.80 | 0.62–1.03 | 0.099 |
| Occupational physical activity | | | | | | |
| Low | 148 | 7180 | 71 669 | 1.00 | Reference | |
| High | 116 | 6172 | 62 166 | 1.04 | 0.82–1.32 | |
| Pulse rate (beats/min) | | | | | | |
| ≤72 | 322 | 12 103 | 114 608 | 1.00 | Reference | |
| >72 | 307 | 10 334 | 95 104 | 1.05 | 0.89–1.23 | |
| Systolic blood pressure (mmHg) | | | | | | |
| ≤141 | 227 | 11 366 | 111 257 | 1.00 | Reference | |
| >141 | 402 | 11 068 | 98 447 | 0.98 | 0.82–1.16 | |
| History of diabetes | | | | | | |
| No | 604 | 21 921 | 206 337 | 1.00 | Reference | |
| Yes | 37 | 855 | 5749 | 1.31 | 0.93–1.82 | |
| Blood glucose levels among non-diabetics (mmol/l) | | | | | | |
| <8.0 | 556 | 19 071 | 178 418 | 1.00 | Reference | |
| ≥8.0 | 37 | 967 | 8626 | 1.15 | 0.82–1.61 | |

[a]Information on each variable was not available on all participants. [b]Two-sided *P*-values for trend by proportional hazards model when variables were treated as ordinal variables.

**Table 4** Age-adjusted relative risks (RRs) and 95% confidence intervals (CIs) of incident prostate cancer associated with marital status, education and occupation in a cohort of 22 895 Norwegian men

| Variable[a] | No. of cases | No. of men | No. of person-years | RR | 95% CI | *P* for trend[b] |
|---|---|---|---|---|---|---|
| Marital status | | | | | | |
| Married | 487 | 18 070 | 172 230 | 1.00 | Reference | |
| Unmarried | 70 | 2631 | 23 485 | 0.94 | 0.73–1.21 | |
| Widower | 68 | 1380 | 9364 | 0.98 | 0.75–1.29 | |
| Divorced/separated | 19 | 760 | 7127 | 1.56 | 0.97–2.44 | |
| Educational attainment | | | | | | |
| Primary or lower secondary (0–9 years) | 435 | 13 505 | 122 994 | 1.00 | Reference | |
| Upper secondary (10–12 years) | 80 | 3355 | 33 555 | 1.18 | 0.92–1.50 | |
| College or university (13+ years) | 37 | 1602 | 16 216 | 1.56 | 1.11–2.19 | 0.007 |
| Occupation | | | | | | |
| Unemployed, unskilled, fishermen, subordinate staff | 137 | 5021 | 46 516 | 1.00 | Reference | |
| Farmers | 150 | 4517 | 41 694 | 1.09 | 0.86–1.37 | |
| Skilled, professional/management, self-employed | 218 | 8112 | 79 021 | 1.30 | 1.05–1.61 | |

[a]Information on each variable was not available on all participants. [b]Two-sided *P*-values for trend by proportional hazards model when variables were treated as ordinal variables.

occupations of lower status (RR = 1.30; 95% CI 1.05–1.61). These results were not altered after adjustment for potentially confounding variables (data not shown).

To explore whether the results of the study could be biased due to differential testing with PSA between different categories of exposure, we used 1 January 1993 as cut-off date for follow-up. These analyses included 460 cases of prostate cancer diagnosed during a mean follow-up of 7.1 years, and mean age at diagnosis was 75.6 years. However, the estimates of relative risks were similar to those obtained with full follow-up (data not shown).

We also restricted the analysis to metastatic prostate cancer (data not shown), and found a similar increase in risk associated with socio-economic status, but confidence limits were wide due to fewer cases in each category. Moreover, the indications of a negative association with leisure-time physical activity persisted in the analysis of metastatic disease. The RR for highly active men was 0.65 (95% CI 0.40–1.06) compared with the least active men, and the RR for men with a high frequency/intensity/duration-index was 0.65 (95% CI 0.39–1.09) compared with men with a low index value.

© 2000 Cancer Research Campaign 

### DISCUSSION

In this 12-year follow-up of 22 895 Norwegian men 40 years and older, we found that men with high socio-economic status (i.e. long education and/or employed in occupations of high socio-economic status) had an elevated risk of prostate cancer. This is in agreement with some studies (Rimpelä and Pukkala, 1987; Yu et al, 1988; Williams et al, 1991; Harvei and Kravdal, 1997), contradictory to others (Oishi et al, 1989; Fincham et al, 1990), while a few have reported no association (Talamini et al, 1986; Severson et al, 1989). We also found that divorced or separated men might have a higher risk of prostate cancer than married men. Epidemiological data on the association between marital status and prostate cancer are inconsistent (Newell et al, 1987; Yu et al, 1988; Severson et al, 1989; Hayes et al, 1992; La Veccia et al, 1993; Harvei and Kravdal, 1997).

It is conceivable that some of these results could be biased by differential PSA testing between categories of the cohort. However, when we restricted our analysis to the period before PSA screening became prevalent in Norway, using 1 January 1993 as cut-off for follow-up, there was no material change in the estimates of relative risk. Another possibility that we cannot rule out is a higher incidence among men with high socio-economic status due to greater medical attention. The frequency of TURPs (transurethral resection of the prostate) might also be higher in this group, and thus influence the prostate cancer detection rate. However, neither a strong positive association with age, nor a pronounced international and racial variation is likely to disturb the results of our study. First, our estimates were adjusted for age (across 2.5-year categories), and second, inhabitants in this part of Norway constitute a population of homogenous ethnicity; hence, very few people are non-Caucasian.

Sexual factors have been suggested to have bearing on the development of prostate cancer (Rotkin, 1977; Ross et al, 1987; Oishi et al, 1990, La Veccia et al, 1993), partly because sexual activity might mirror the endocrine milieu of androgens, and partly because men with an active sexual life have more chances to be exposed to transmittable oncogenic agents. Although marital status probably is not a good indicator of sexual activity, the increased risk seen among divorced and separated men in our study could be explained by such mechanisms. Moreover, the higher educated may have more sexual partners than those with less education (Binson et al, 1993). A higher risk of prostate cancer has been found among men in occupations that involve travelling (Pearce et al, 1987), and their sexual activity may also include several partners (National Institute of Public Health, 1993).

We found a weak positive association with number of cigarettes, which is in agreement with some studies (Hsing et al, 1991; Cerhan et al, 1997), while others have reported no association (Adami et al, 1996, Lumey et al, 1997). It has been reported that certain nitrosamines may induce prostate cancer in laboratory rats (Pour, 1983), but an association could also be mediated through the endocrine pathway, since smoking appears to increase levels of circulating androgens in men (Dai et al, 1988).

Available epidemiological data do not indicate a causal role of alcohol (Breslow and Weed, 1998), and our findings are consistent with this. However, our data suggest a slightly increased risk among teetotallers compared to drinking men, but biological mechanisms explaining such an association are not evident, although animal studies show that alcohol may decrease plasma testosterone (Badr and Bartke, 1974).

We also found suggestive evidence of a negative association between physical exercise and risk of prostate cancer, and this association persisted in the analysis of metastatic disease. Physical activity may reduce levels of circulating testosterone (Aakvaag et al, 1978; Hackney et al, 1988), but previous findings have been inconsistent (Le Marchand et al, 1991; Lee et al, 1992; Thune and Lund, 1994; Hartman et al, 1998). Our measures of pulse rate and blood pressure could, in addition to mirroring levels of physical activity, serve as surrogate markers of sympathetic nervous activity, which may influence prostatic growth (Wang et al, 1991). We found, however, no association with these physiological variables, although previous epidemiological data indicate a relation (Gann et al, 1995).

Previously, a lower risk of prostate cancer has been suggested for men with diabetes mellitus (Thompson et al, 1989; Giovannucci et al, 1998). However, we found a non-significantly higher risk of prostate cancer among men with diabetes in our study, a finding that is supported by Steenland et al (1995). In addition, we hypothesized that high levels of blood glucose among men without diabetes could identify men with lower risk of prostate cancer, but found no association between levels of non-fasting blood glucose and prostate cancer among non-diabetic men.

In summary, our study suggests that men of higher socio-economic status have increased risk of prostate cancer, and that divorced men might be at higher risk of developing the disease. Furthermore, number of cigarettes smoked per day showed a weak positive association with prostate cancer, while men reporting high physical activity may have had a reduced risk of prostate cancer.

### ACKNOWLEDGEMENTS

The research is based on data made available by the National Health Screening Service, The Cancer Registry of Norway, and the National Institute of Public Health, Community Medicine Research Centre in Verdal, Nord-Trøndelag County, Norway. The project was supported by grant E 98010 to Lund Nilsen from the Norwegian Cancer Society, Oslo, Norway.

### REFERENCES

Aakvaag A, Sand T, Opstad PK and Fonnum F (1978) Hormonal changes in serum in young men during prolonged physical strain. *Eur J Appl Physiol* **39:** 283–291

Adami HO, Bergstrom R, Engholm G, Nyren O, Wolk A, Ekbom A, Englund A and Baron J (1996) A prospective study of smoking and risk of prostate cancer. *Int J Cancer* **67:** 764–768

Andersson SO, Baron J, Bergstrom R, Lindgren C, Wolk A and Adami HO (1996) Lifestyle factors and prostate cancer risk: a case–control study in Sweden. *Cancer Epidemiol Biomarkers Prev* **5**: 509–513

Badr FM and Bartke A (1974) Effect of ethyl alcohol on plasma testosterone level in mice. *Steroids* **23**: 921–928

Binson D, Dolcini MM, Pollack LM and Catania JA (1993) Data from the National AIDS Behavioral Surveys. IV. Multiple sexual partners among young adults in high-risk cities. *Fam Plann Perspect* **25**: 268–272

Breslow RA and Weed DL (1998) Review of epidemiologic studies of alcohol and prostate cancer: 1971–1996. *Nutr Cancer* **30**: 1–13

Cancer Registry of Norway (1998). *Cancer in Norway 1995.* The Cancer Registry of Norway: Oslo

*© 2000 Cancer Research Campaign*

Cerhan JR, Torner JC, Lynch CF, Rubenstein LM, Lemke JH, Cohen MB, Lubaroff DM and Wallace RB (1997) Association of smoking, body mass, and physical activity with risk of prostate cancer in the Iowa 65+ Rural Health Study (United States). *Cancer Causes Control* **8**: 229–238

Colditz G (1996) Consensus conference: smoking and prostate cancer. *Cancer Causes Control* **7**: 560–562

Dai WS, Gutai JP, Kuller LH and Cauley JA (1988) Cigarette smoking and serum sex hormones in men. *Am J Epidemiol* **128**: 796–805

Fincham SM, Hill GB, Hanson J and Wijayasinghe C (1990) Epidemiology of prostatic cancer: a case–control study. *Prostate* **17**: 189–206

Gann PH, Daviglus ML, Dyer AR and Stamler J (1995) Heart rate and prostate cancer mortality: results of a prospective analysis. *Cancer Epidemiol Biomarkers Prev* **4**: 611–616

Giovannucci E, Rimm EB, Stampfer MJ, Colditz GA and Willett WC (1998) Diabetes mellitus and risk of prostate cancer. *Cancer Causes Control* **9**: 3–9

Hackney AC, Sinning WE and Bruot BC (1988) Reproductive hormonal profiles of endurance-trained and untrained males. *Med Sci Sports Exerc* **20**: 60–65

Haenszel W and Kurihara M (1968) Studies of Japanese migrants. I. Mortality from cancer and other diseases among Japanese in the United States. *J Natl Cancer Inst* **40**: 43–68

Hartman TJ, Albanes D, Rautalahti M, Tangrea JA, Virtamo J, Stolzenberg R and Taylor PR (1998) Physical activity and prostate cancer in the Alpha-Tocopherol, Beta-Carotene (ATBC) Cancer Prevention Study. *Cancer Causes Control* **9**: 11–18

Harvei S and Kravdal O (1997) The importance of marital and socioeconomic status in incidence and survival of prostate cancer. An analysis of complete Norwegian birth cohorts. *Prev Med* **26**: 623–632

Harvei S, Tretli S and Langmark F (1996) Quality of prostate cancer data in the cancer registry of Norway. *Eur J Cancer* **32A**: 104–110

Hayes RB, de Jong FH, Raatgever J, Bogdanovicz J, Schroeder FH, van der Maas P, Oishi K and Yoshida O (1992) Physical characteristics and factors related to sexual development and behaviour and the risk for prostatic cancer. *Eur J Cancer Prev* **1**: 239–245

Hayes RB, Brown LM, Schoenberg JB, Greenberg RS, Silverman DT, Schwartz AG, Swanson GM, Benichou J, Liff JM, Hoover RN and Pottern LM (1996) Alcohol use and prostate cancer risk in US blacks and whites. *Am J Epidemiol* **143**: 692–697

Hiatt RA, Armstrong MA, Klatsky AL and Sidney S (1994) Alcohol consumption, smoking, and other risk factors and prostate cancer in a large health plan cohort in California (United States). *Cancer Causes Control* **5**: 66–72

Holmen J and Midthjell K (1990) *The Nord-Trøndelag Health Survey 1984–86: Purpose, Background and Methods: Participation, Non-participation and Frequency*. Report no 4, National Institute of Public Health: Oslo

Hsing AW, McLaughlin JK, Hrubec Z, Blot WJ and Fraumeni JF Jr (1991) Tobacco use and prostate cancer: 26-year follow-up of US veterans. *Am J Epidemiol* **133**: 437–441

Kleinbaum DG (1995) *Survival Analysis: a Self-learning Text*. Springer-Verlag: New York

La Vecchia C, Franceschi S, Talamini R, Negri E, Boyle P and D'Avanzo B (1993) Marital status, indicators of sexual activity and prostatic cancer. *J Epidemiol Community Health* **47**: 450–453

Landis SH, Murray T, Bolden S and Wingo PA (1998) Cancer statistics, 1998. *CA Cancer J Clin* **48**: 6–29

Le Marchand L, Kolonel LN and Yoshizawa CN (1991) Lifetime occupational physical activity and prostate cancer risk. *Am J Epidemiol* **133**: 103–111

Lee IM, Paffenbarger RS Jr and Hsieh CC (1992) Physical activity and risk of prostatic cancer among college alumni. *Am J Epidemiol* **135**: 169–179

Lumey LH, Pittman B, Zang EA and Wynder EL (1997) Cigarette smoking and prostate cancer: no relation with six measures of lifetime smoking habits in a large case–control study among U.S. whites. *Prostate* **33**: 195–200

Lumey LH, Pittman B and Wynder EL (1998) Alcohol use and prostate cancer in U.S. whites: no association in a confirmatory study. *Prostate* **36**: 250–255

Muir CS, Nectoux J and Staszewski J (1991) The epidemiology of prostatic cancer. Geographical distribution and time-trends. *Acta Oncol* **30**: 133–140

National Institute of Public Health (1993) *Rapport fra seksualvaneundersøkelsene i 1987 og 1992* [in Norwegian]. National Institute of Public Health: Oslo

Newell GR, Pollack ES, Spitz MR, Sider JG and Fueger JJ (1987) Incidence of prostate cancer and marital status. *J Natl Cancer Inst* **79**: 259–262

Oishi K, Okada K, Yoshida O, Yamabe H, Ohno Y, Hayes RB and Schroeder FH (1989) Case–control study of prostatic cancer in Kyoto, Japan: demographic and some lifestyle risk factors. *Prostate* **14**: 117–122

Oishi K, Okada K, Yoshida O, Yamabe H, Ohno Y, Hayes RB, Schroeder FH and Boyle P (1990) A case–control study of prostatic cancer in Kyoto, Japan: sexual risk factors. *Prostate* **17**: 269–279

Parker SL, Davis KJ, Wingo PA, Ries LA and Heath CW Jr (1998) Cancer statistics by race and ethnicity. *CA Cancer J Clin* **48**: 31–48

Pearce NE, Sheppard RA and Fraser J (1987) Case–control study of occupation and cancer of the prostate in New Zealand. *J Epidemiol Community Health* **41**: 130–132

Pour PM (1983) Prostatic cancer induced in MRC rats by N-nitrosobis(2-oxopropyl)-amine and N-nitrosobis(2-hydroxypropyl)amine. *Carcinogenesis* **4**: 49–55

Rimpelä AH and Pukkala EI (1987) Cancers of affluence: positive social class gradient and rising incidence trend in some cancer forms. *Soc Sci Med* **24**: 601–606

Ross RK, Shimizu H, Paganini-Hill A, Honda G and Henderson BE (1987) Case–control studies of prostate cancer in blacks and whites in southern California. *J Natl Cancer Inst* **78**: 869–874

Rotkin ID (1977) Studies in the epidemiology of prostatic cancer: expanded sampling. *Cancer Treat Rep* **61**: 173–180

Severson RK, Nomura AM, Grove JS and Stemmermann GN (1989) A prospective study of demographics, diet, and prostate cancer among men of Japanese ancestry in Hawaii. *Cancer Res* **49**: 1857–1860

Steenland K, Nowlin S and Palu S (1995) Cancer incidence in the National Health and Nutrition Survey I. Follow-up data: diabetes, cholesterol, pulse and physical activity. *Cancer Epidemiol Biomarkers Prev* **4**: 807–811

Talamini R, La Vecchia C, Decarli A, Negri E and Franceschi S (1986) Nutrition, social factors and prostatic cancer in a Northern Italian population. *Br J Cancer* **53**: 817–821

Thompson MM, Garland C, Barrett-Connor E, Khaw KT, Friedlander NJ and Wingard DL (1989) Heart disease risk factors, diabetes, and prostatic cancer in an adult community. *Am J Epidemiol* **129**: 511–517

Thune I and Lund E (1994) Physical activity and the risk of prostate and testicular cancer: a cohort study of 53 000 Norwegian men. *Cancer Causes Control* **5**: 549–556

Wang JM, McKenna KE, McVary KT and Lee C (1991) Requirement of innervation for maintenance of structural and functional integrity in the rat prostate. *Biol Reprod* **44**: 1171–1176

WHO (1980) *WHO expert committee on diabetes mellitus, Second report*. Technical report series no 646. World Health Organization: Geneva

Williams J, Clifford C, Hopper J and Giles G (1991) Socio-economic status and cancer mortality and incidence in Melbourne. *Eur J Cancer* **27**: 917–921

Yu H, Harris RE and Wynder EL (1988) Case–control study of prostate cancer and socioeconomic factors. *Prostate* **13**: 317–325

*© 2000 Cancer Research Campaign*
